# Cytotoxic and Insecticidal Activities of Derivatives of Harmine, a Natural Insecticidal Component Isolated from *Peganum harmala*

**DOI:** 10.3390/molecules15117775

**Published:** 2010-11-02

**Authors:** Yong Zeng, Yaomou Zhang, Qunfang Weng, Meiying Hu, Guohua Zhong

**Affiliations:** 1 Laboratory of Insect Toxicology, South China Agricultural University, Guangzhou 510642, China; E-Mails: yongz800@163.com (Y.Z.); huabao@scau.edu.cn (Q.F.W); humy@scau.edu.cn (M.Y.H.); 2 College of Science, South China Agricultural University, Guangzhou 510642, China; E-Mail: zhangyaom@163.com (Y.Z.)

**Keywords:** harmine, synthesis, derivatives, cytotoxicity, insecticidal activity

## Abstract

In a continuing effort to develop novel β-carbolines endowed with better insecticidal activity, a simple high-yielding method for the synthesis of harmine compounds starting from L-tryptophan has been developed and a series of 1,3-substituted β-carboline derivatives have been synthesized and evaluated for their cytotoxicity against insect cultured Sf9 cell line in *vitro* and insecticidal activities against 4th instar larvae of mosquitos, *Culex pipiens quinquefasciatus* and mustard aphid, *Lipaphis erysimi*. The results demonstrated that 1-phenyl-1,2,3,4-tetrahydro-β-carboline-3-carboxylic acid (compound **2**) and methyl 1-phenyl-β-carboline-3-carboxylate (compound **13**) represented the best potential compounds, with Sf9 cells inhibition rates of 71.55% and 60.21% after 24 h treatment at concentrations of 50–200 mg/L, respectively. Both compounds **2** and **13** also showed strong insecticidal activity towards 4th instar larvae of mosquitos with LC_50_ values of 20.82 mg/L and 23.98 mg/L, and their LC_90_ values were 88.29 mg/L and 295.13 mg/L, respectively. Furthermore, the LC_50_ values of compounds **2** and **13** against mustard aphids were 53.16 mg/L and 68.05 mg/L, and their LC_90_ values were 240.10 mg/L and 418.63 mg/L after 48 h treatment. The *in vitro* cytotoxicity of these compounds was consistent with the insecticidal activity *in vivo*. The results indicated that the 1- and 3-positions of the β-carboline ring deserve further investigation to develop biorational insecticides based on the natural compound harmine as a lead compound.

## 1. Introduction

Harmine compounds and structurally related compounds, belonging to the β-carboline alkaloids, and found in medicinal plants such as *Peganum harmala* and *Eurycoma longifolia*, have recently drawn increasing interest due to their diverse pharmacological, neurophysiologic and biochemical activities [1,2,3,4,5,6]. Some plants which contain these compounds are used in traditional medicine in China, Brazil and other parts of the world for their emmenagogue, abortifacient, anticancer, antispasmodic and sedative properties [7,8]. 

As for pest management *Peganum* L. plant species extracts, whose main components include a mixture of harmine, harmaline, norharman and their derivatives, have been long known to have insecticidal, fungicidal and plant growth regulatory properties [9,10,11,12,13,14,15]. After treatment with harmaline, an important component of *P. harmala*, Indian meal moth (*Plodia interpunctella*) larvae growth and development was inhibited [16]. When incorporated into the diet, harmaline caused larval weight loss with a reduction in protein and glycogen contents and an inhibition of α-amylase activity. Using electron microscopy, harmaline was shown to cause severe cytotoxicity on the epithelial cells of the midgut, resulting in marked vacuolization of the cytoplasm, the appearance of numerous autophagic vesicles and lysosomic structures, fragmentation of rough endoplasmic reticulum cisternae, disruption of microvilli, and rupture of the plasma membrane leading to shedding of the cytoplasm contents into the midgut lumen. Another study revealed an elevated juvenile hormone epoxide hydrolase (JHEH) activity stimulated by norharmane by accelerating juvenile hormone metabolism in *Reticulitermes speratus* and forcing termite caste differentiation in workers, nymphs, and ergatoids [17]. Our investigations showed that a methanolic extract of *P. harmala* at a concentration of 10,000 mg/L caused mortality rates of 93.07% and 96.36%, respectively, against *Lipaphis erysimi* 24 h and 48 h after treatment [18]. Nematocidal toxicity tests showed that harmine was one of the main nematocidal components of *P. harmala* and its LC_50_ value against mixed instar *B. xylophilus* was 135.74 mg/L after 48 h [11]. Further research demonstrated that harmaline and other active substances of *P. harmala* could disturb normal physiological function, for example, in the 4th instar larvae of *Spodoptera litura,* the haemolymph protein content was significantly reduced and the total body sugar was obviously reduced when treated with the 12th and 14th fractions at 500 mg/L [19]. The latest research showed that after treating with harmaline several growth and development related enzymes of *Noctuidae* Lepidoptera changed regularly [15]. All research showed that harmine compounds could be used as novel insect growth and development inhibitors. The structural simplicity of β-carboline alkaloids hides a multitude of *in vitro* and *in vivo* effects and makes these molecules interesting from both a biophysical and a medicinal perspective. Some such compounds have already been synthesized, however, no systematical pesticidal activity has been reported yet. This paper describes work aimed at preparing a series of new carboline derivatives that might possesses cytotoxic and insecticidal activity. A cultured Sf9 insect cell line from *S. frugiperda* was used for primary screening, followed by the treatment of larvae and adults to determine insecticidal activity. The purpose of this study is to find the new lead compounds and evaluate the prospects of these compounds for practical use in agriculture.

## 2 Results and Discussion

### 2.1. Preparation of compounds

In this paper, five tetrahydro-β-carboline carboxylic acids **1-5** were produced by Pictet–Spengler reaction of L-tryptophan with five different aldehydes (Table 1). Esterification reactions of the tetrahydro-β-carboline carboxylic acid group at position 3 with methanol led to the five desired methyl ester **6-10 in** reasonable yields (Table 1). Oxidative dehydrogenation of methyl tetrahydro-β-carbolines with potassium permanganate as oxidant was also carried out to prepare five β-carboline carboxylates **11-15** (Table 2). The β-carboline carboxylate **11** was hydrolysed using sodium hydroxide in ethanol to give **16** in good yield (Table 2). All compounds were characterised by ^1^H-NMR. 

Harmine compounds as multi-active chemicals could endogenously form from tryptophan and tryptophan-derived indoleamines in alkaloid biosynthesis in mammals and plants [20]. Furthermore, they can be produced from tryptophan residues during conventional high-temperature cooking [21]. In present study, most of new structure β-carboline derivatives, tetrahydro-β-carboline and β-carboline compounds were synthesized using the Pictet–Spengler and Bischler-Napieralski methods [22]. The tetrahydro-β-carboline derivatives were obtained using different mole ratios of aldehydes to tryptophan in the presence of a catalyst. As the literature reports, the yields of these reactions were determined by the reaction conditions and the structures of the aldehydes used. Aldehydes with an electron-withdrawing group underwent *endo* cyclization with tryptophan methyl ester in good yields in water, while the aldehydes bearing electron-donating groups need a polar organic solvent [23]. β-Carboline derivatives were obtained by reaction of a tetrahydro-β-carboline with an oxidant such as sulfur in refluxing xylene, KMnO_4_ in DMF, *etc*. [8]. There are also numerous reports concerning the use of metal complexes as catalysts to generate dihydro-β-carboline or β-carboline alkaloids [24]. The oxidation with sulfur in refluxing xylene would cause environmental pollution because of the organic solvent. Other catalysts usually involve rare metals, which would increase costd. KMnO_4_ as oxidant is very cheap and available, and the reaction can take place under mild conditions, so in this paper, appropriate amounts of KMnO_4_ were added to DMF solutions of tetrahydro-β-carboline-3-carboxylate to produce 1-substituted-β-carboline-3-carboxylates under mild conditions.

### 2.2. Biological activity

#### 2.2.1. Cytotoxicity

**Figure 1:**
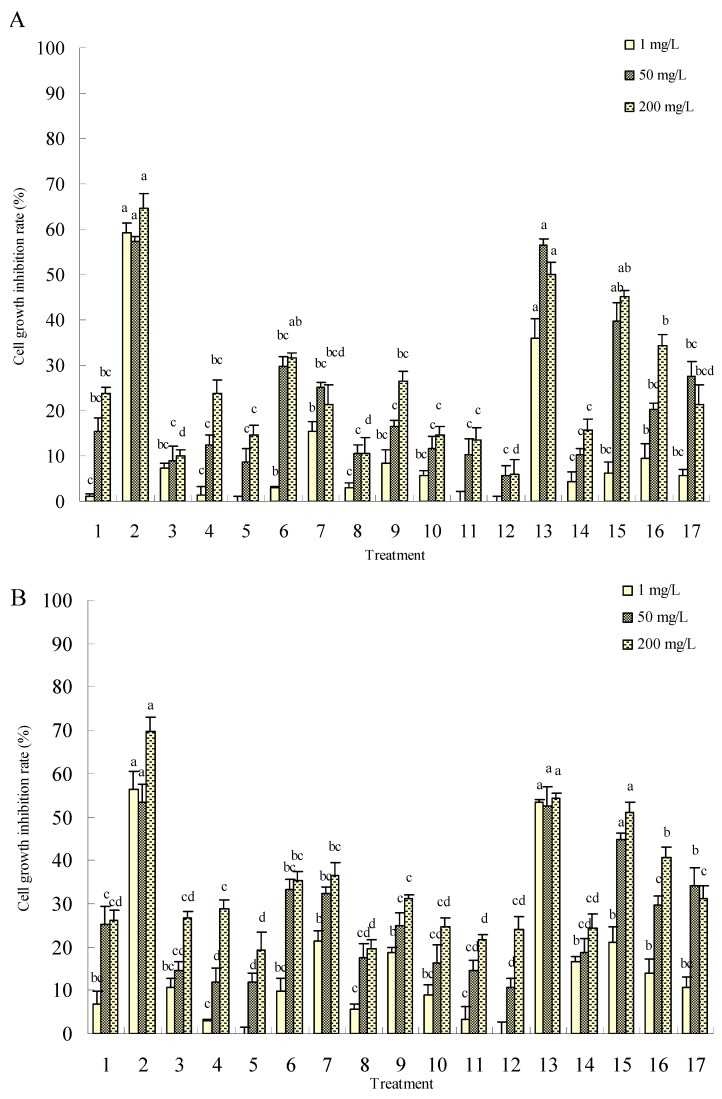

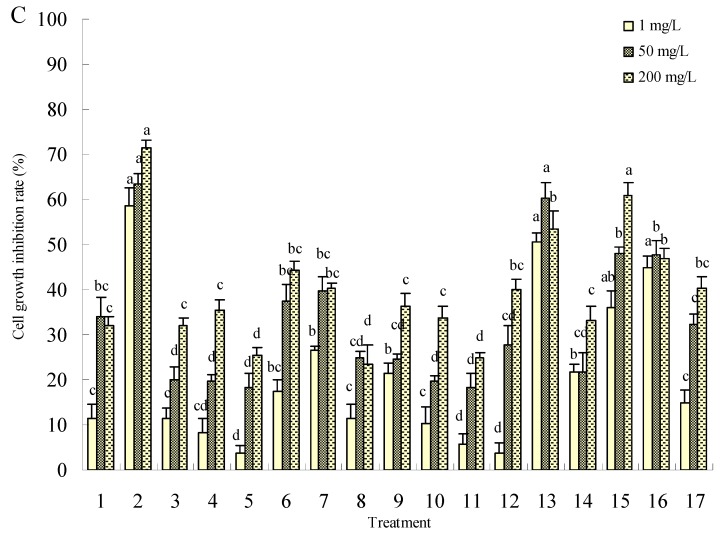
Cytotoxic effect of compounds **1-16** in insect cell line Sf9 determined by the MTT method. A is the effect of 6 h treatment; B is the effect of 12 h treatment; C is the effect of 24 h treatment. Treatments **1-16** are the synthesized compounds and treatment **17** is harmine, a positive control. The error bars represent mean±SEM for data derived from three independent experiments. Mean values followed by the same letter above bars have no significant difference (P ≤ 0.05) according to Duncan’s multiple-range test.

The effects of the synthesized harmaline derivatives on the cytotoxicity of Sf9 cells were examined first. Cells were treated with various concentrations for the indicated time periods (6, 12 and 24 h) and cell cytotoxicity was then assessed using the MTT colorimetric assay. The differences of cytotoxicities on Sf9 cells when treated with different derivatives were statistically significant at the concentrations of 1, 50 and 200 mg/L. The compounds **2**, **13** and **15** showed more cytotoxic effects and caused cell growth inhibition rates of up to 71.55%, 53.42% and 60.86%, respectively, after 24 h treatment at the concentration of 200 mg/L, while the maximum inhibition using harmine treatment as a positive control was only 40.31% under the same conditions (Figure 1). Interestingly, harmine and some of its derivatives exhibited significant differences between antitumor cytotoxicity and insect cell lethal toxicity to some degree. 

For example, 1-trichloromethyl-1,2,3,4-tetrahydro-β-carboline has been reported as an inducer of cell-free plasmid DNA damage [8,25], however, methyl 1-trichloromethyl-1,2,3,4-tetrahydro- β-carboline-3-carboxylate (compound **9**) did not show high cytotoxicity towards Sf9 cells in the present tests, even though both compounds have similar chemical structures, which maybe results from the existence of different targets.

#### 2.2.2. Insecticidal activity

**Figure 2:**
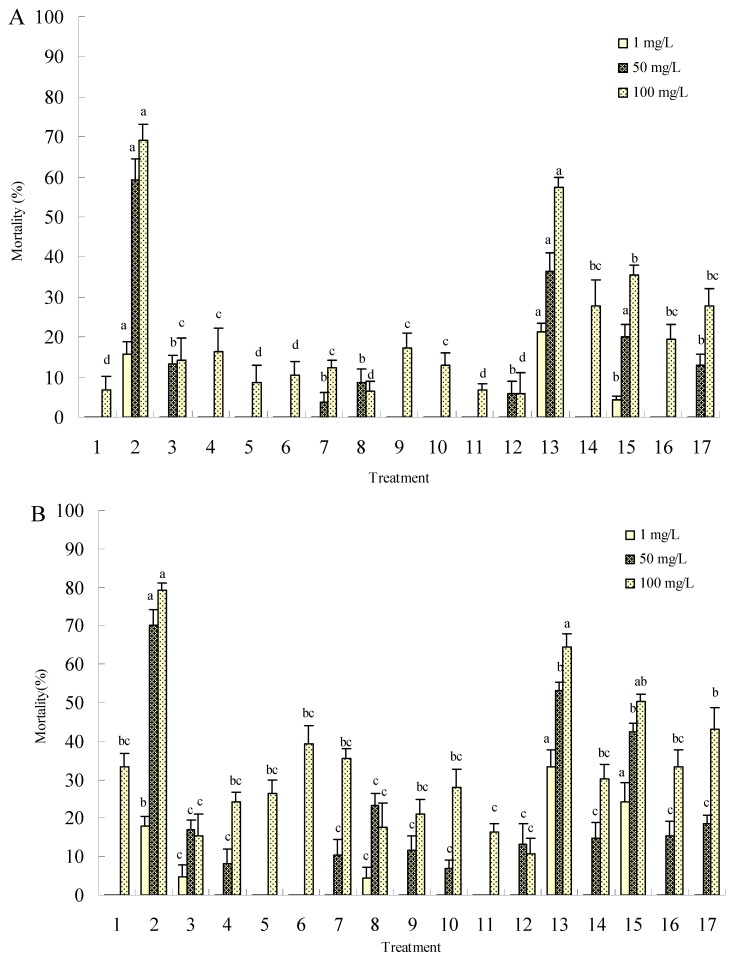

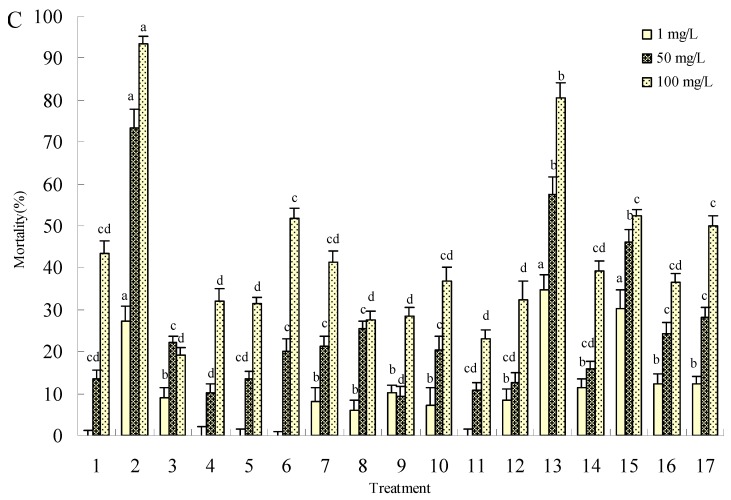
Mosquitocidal effect of compounds **1-16** against 4th instar mosquito (*Cx. pipiens quinquefasciatus*) larvae. A is the effect of 2 h treatment; B is the effect of 12 h treatment; C is the effect of 24 h treatment. Treatments **1-16** are the synthesized compounds and treatment **17** is harmine, a positive control. The error bars represent mean±SEM for data derived from three independent experiments. Mean values followed by the same letter above bars have no significant difference (P ≤ 0.05) according to Duncan’s multiple-range test.

As shown in Figure 2, treatments of 4th instar mosquito larvae with compounds **1-16** resulted in a marked dose-dependent toxicity. All the mortalities were less than 34.85% after treatment at a low concentration of 1 mg/L for a short period of time (24 h), and were not statistically significant. However, compounds **2** and **13** showed stronger toxicity to mosquitos than the other compounds after treatments with both 50 mg/L and 100 mg/L. After 24 h of treatment with 100 mg/L, compounds **2** and **13** gave adjusted mortalities of 93.29% and 80.49%, respectively, against the tested mosquito larvae, thus exhibiting excellent prospects for follow-up. The LC_50_ values of compounds **2** and **13** were 20.82 mg/L and 23.98 mg/L, and the LC_90_ values were 88.29 mg/L and 295.13 mg/L, respectively, after 24 h treatment with the probit (mortality%)-log (concentration) line (Y = 2.3096 + 2.0404×) and (Y = 3.3802 + 1.1740×). 

**Figure 3:**
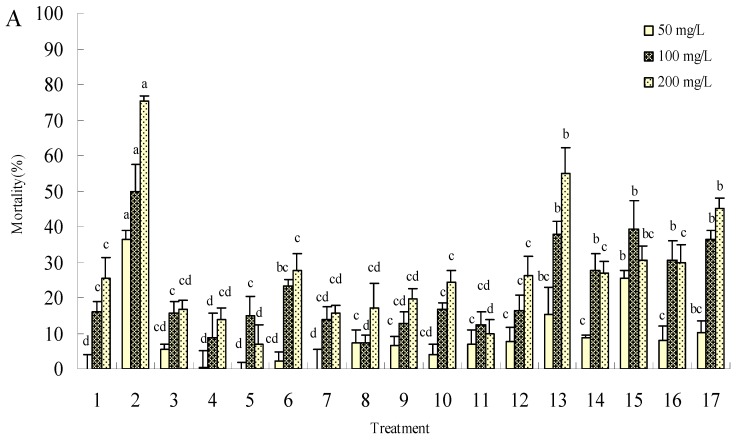

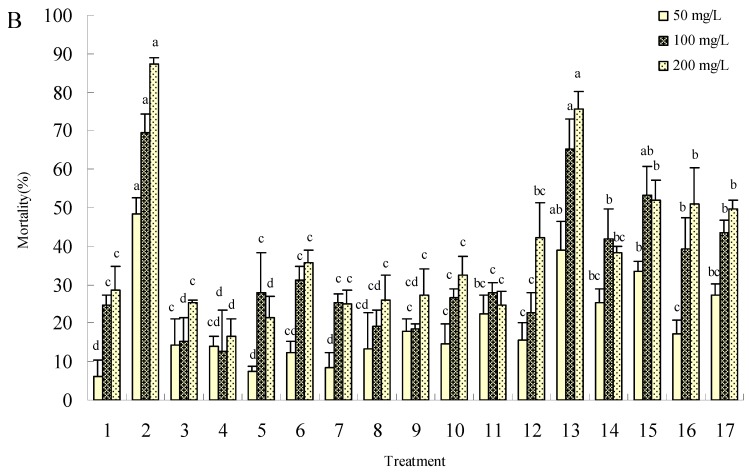
Toxic effect of compounds **1-16** on mustard aphids (*L. erysimi*). A is the effect of 24 h treatment; B is the effect of 48 h treatment. Treatments **1-16** are the synthesized compounds and treatment **17** is harmine, a positive control. The error bars represent mean±SEM for data derived from three independent experiments. Mean values followed by the same letter above bars have no significant difference (P ≤ 0.05) according to Duncan’s multiple-range test.

The mustard aphid mortality increased with oncentration after 24 h and 48 h treatment with 50, 100 and 200 mg/L of the synthesized harmine derivatives, as shown in Figure 3. 

Both compounds **2** and **13** showed the strongest insecticidal activities. The mortalities were only 36.42% and 15.32% after 24 h treatment with compounds **2** and **13** at the concentration of 50 mg/L, but they increased to 87.26% and 75.76%, respectively, after 48 h at 200 mg/L, with a significant difference from treatment with harmine alone (49.62% adjusted mortality) and CK. The LC_50_ values for compounds **2** and **13** were 53.16 mg/L and 68.05 mg/L, and their corresponding LC_90_ values against mustard aphids were 240.10 mg/L and 418.63 mg/L, respectively, after 48 h treatment, as determined in the following equation with probit (mortality%)-log (concentration) line: Y = 1.6268 + 1.9548×, Y = 2.0266 + 1.6223×, The results were in line with the cytotoxicity and mosquitocidal toxicity experiments, and suggest these compounds could be used can be used as candidate compounds to guide further research.

## 3. Experimental

### 3.1. General procedures

Solvents and reagents were obtained from commercial sources and purified according to the usual procedures described in the literature [26]. Progress of the reaction and purity of the compounds were monitored by thin-layer chromatography (TLC). ^1^H-NMR spectra were recorded on a Bruker Avance-600 superconducting nuclear magnetic resonance instrument, with chemical shift (δ) values reported in ppm relative to tetramethylsilane (TMS) as internal standard. Melting points were determined on an XT-4 binocular microscope (Beijing Tech Instrument Co., Beijing, China) and are uncorrected. Bioassays were carried out under standard lab conditions. 

### 3.2. Chemical synthesis

*1-Methyl-1,2,3,4-tetrahydro-β-carboline-3-carboxylic acid* (**1**): Acetaldehyde (40%, 1.1 mL) was added to a solution of L-tryptophan (2.0 g, 10 mmol) in 0.1M aqueous hydrochloric acid (10 mL). This reaction mixture was stirred at room temperature for 12 h. The resulting crystals were filtered, dissolved in hot water and the hot solution was adjusted to pH 8 with aqueous sodium hydroxide solution. Upon cooling, the product [27,28,29] was collected as white needle-like crystals in 90% yield, mp, >200 °C; ^1^H-NMR (DMSO-d_6_) δ: 1.25 (3H, d, *J =* 7.8 Hz, CH_3_), 2.87 (1H, dd, *J* = 6.6 Hz, C(4)H, c), 3.19 (1H, dd, *J* = 4.8 Hz, C(4)H, c), 3.51 (1H, bs, N(2)H, c), 4.31 (1H, q, *J* = 7.6 Hz, C(1)H, c), 4.95 (1H, q, *J* = 6.6 Hz, C(3)H, c), 7.08~7.72 (4H, m, C(5,6,7,8)H, c), 9.35 (1H, bs, N(9)H, c), 10.30 (1H, s, COOH). 

*1-Phenyl-1,2,3,4-tetrahydro-β-carboline-3-carboxylic acid* (**2**): L-Tryptophan (2.0 g, 10 mmol) was dissolved in methanol (10 mL) which contain hydrochloric acid (1 mL), and then benzaldehyde (2 mL) was added to the solution. This reaction mixture was heated to reflux for 2 h. The resulting mixture was cooled to room temperature, poured into water and adjusted to pH 8 with aqueous sodium hydroxide solution [30]. The precipitate was filtered and dissolved in a hot mixture of 5mL methanol and 8mL ethyl acetate. Upon cooling, the product was collected in 82.4% yield as a white powder, mp, 174–176 °C; ^1^H-NMR (DMSO-d_6_): δ 2.24 (1H, bs, N(2)H, c), 3.00 (1H, dd, *J* = 4.8 Hz, C(4)H, c), 3.23 (1H, dd, *J* = 8.4 Hz, C(4)H, c), 4.09 (1H, q, *J* = 7.6 Hz, C(3)H, c), 5.61 (1H, q, *J* = 5.2 Hz, C(1)H, c), 6.99~7.49 (4H, m, C(5,6,7,8)H, c), 7.45 (4H, s, C(2,3,5,6)H, Ph), 9.24 (1H, bs, N(9)H, c), 10.60 (1H, s, COOH).

*1,2,3,4-Tetrahydro-β-carboline-3-carboxylic acid* (**3**): A mixture of methanal (30%, 1 mL) and L-tryptophan (2.0 g, 10 mmol) in acetic acid (5 mL) was heated to reflux for 6 h or stirred at room temperature for 24 h. The precipitate was filtered and recrystallized from hot methanol, in 40.1% yield, mp, >200 °C. ^1^H-NMR (DMSO-d_6_): δ 1.88 (1H, bs, N(2)H, c), 2.76 (1H, dd, *J* = 5.2 Hz, C(4)H, c), 3.06 (1H, dd, *J* = 6.8 Hz, C(4)H, c), 4.23 (1H, d, *J =* 7.8 Hz, C(1)H, c), 4.38 (1H, d, *J =* 10.2 Hz, C(1)H, c), 5.39 (1H, q, *J* = 8.4 Hz, C(3)H, c), 7.03 ~7.48 (4H, m, C(5,6,7,8)H, c), 8.80 (1H, bs, N(9)H, c), 11.24 (1H, s, COOH).

*1-(Trichloromethyl)-1,2,3,4-tetrahydro-β-carboline-3-carboxylic acid* (**4**): The method used to prepare this compound was as described in 3.2.3. L-Tryptophan (2.0 g) and trichloroacetaldehyde monohydrate (1.65 g) were reacted at reflux temperature, and finally collected in crystalline form in 32.6% yield, mp > 200 °C. The crystals readily changed color in air. ^1^H-NMR (DMSO-d_6_): δ 1.92 (1H, bs, N(2)H, c), 2.96 (1H, dd, *J* = 6.8 Hz, C(4)H, c), 3.09 (1H, dd, *J* = 8.4 Hz, C(4)H, c), 4.29 (1H, q, *J* = 10.8Hz, C(3)H, c), 5.11 (1H, q, *J* = 7.2 Hz, C(1)H, c), 6.99~7.48 (4H, m, C(5,6,7,8)H, c), 9.28 (1H, bs, N(9)H, c), 10.78 (1H, s, COOH).

*1-(4-Hydroxy-3-methoxyphenyl)-1,2,3,4-tetrahydro-β-carboline-3-carboxylic acid* (**5**): The method described in 3.2.3 was used to prepare the title compound. L-Tryptophan (2.0 g) and vanillic aldehyde (1.5g) with 1:1 molar quantities were added to a round-bottom flask and stirred at room temperature for 24 h. A large amount of a yellowish green solid was deposited on the bottom of the flask. The target compound was isolated in 77.8% yield by filtering the mixture, mp, 184–186 °C; ^1^H-NMR (DMSO-d_6_): δ 1.91 (1H, bs, N(2)H, c), 2.93 (1H, dd, *J* = 8.4 Hz, C(4)H, c), 3.11 (1H, q, *J* = 7.8 Hz, C(4)H, c), 3.65 (1H, q, *J* = 5.2 Hz, C(3)H, c), 3.74 (3H, s, Ar-OCH_3_), 5.47 (1H, q, *J* = 6.6 Hz, C(1)H, c), 6.50~6.71 (2H, m, C(5,6)H, Ph), 7.01 (1H, s, C(2)H, Ph), 6.97~7.46 (4H, m, C(5,6,7,8)H, c), 9.32 (1H, bs, N(9)H, c), 10.69 (1H, s, COOH).

*Methyl 1-methyl-1,2,3,4-tetrahydro-β-carboline-3-carboxylate* (**6**): The synthesis of this compound was accomplished by a two-step process. Methanol (15 mL) was cooled to 0 °C and thionyl chloride (1 mL) was added dropwise. Fifteen min later, the cooling bath was removed and compound **1** was added to the solution. The mixture was stirred overnight followed by distillation under reduced pressure and the residue was adjusted to pH 8 with aqueous sodium hydroxide solution [29]. The deposits were filtered and re-crystallized from hot methanol (3 mL) with the addition of ethyl acetate (8 mL) to give a 85.9% yield of the target compound, mp, 185–187 °C; ^1^H-NMR (DMSO-d_6_): δ 1.72 (3H, d, *J =* 6.6 Hz, CH_3_, c), 2.50 (1H, bs, N(2)H, c), 3.02 (1H, dd, *J* = 11.2 Hz, C(4)H, c), 3.26 (1H, dd, *J* = 6.4 Hz, C(4)H, c), 3.86 (3H, s, COOCH_3_), 4.58 (1H, q, *J*= 8.2 Hz, C(3)H, c), 4.72 (1H, q, *J* = 6.0 Hz, C(1)H, c), 7.02~7.48 (4H, m, C(5,6,7,8)H, c), 11.36 (1H, bs, N(9)H, c).

*Methyl 1-phenyl-1,2,3,4-tetrahydro-β-carboline-3-carboxylate* (**7**): In a round-bottomed, short-necked flask crude methyl 2-amino-3-(1*H*)-indol-3-yl propanoate (2.55 g, 10 mmol) was dissolved in methanol (10 mL) and benzaldehyde (1 g, 10 mmol) was added to the mixture with stirring. The resulting solution was boiled for 6 h under reflux. The excess methanol was distilled off under reduced pressure and the residue was adjusted to pH 8 with aqueous sodium hydroxide solution [30]. The deposits were filtered and re-crystallized from hot methanol (1 mL) with the addition of ethyl acetate (5 mL), to give a 87.2% yield of compound **7**, mp, >200 °C; ^1^H-NMR (DMSO-d_6_): δ 2.25 (1H, bs, N(2)H, c), 2.99 (1H, dd, *J* = 8.8 Hz, C(4)H, c), 3.21 (1H, dd, *J* = 4.6 Hz, C(4)H, c), 3.80 (3H, s, COOCH_3_), 3.96 (1H, q, *J* = 10.2 Hz, C(3)H, c), 5.22 (1H, q, *J* = 8.2 Hz, C(1)H, c), 7.10~7.53 (4H, m, C(5,6,7,8)H, c), 7.18~7.36 (5H, s, C(2,3,4,5,6)H, Ph), 11.13 (1H, s, N(9)H, c).

*Methyl 1-(4-hydroxy-3-methoxyphenyl)-1,2,3,4-tetrahydro-β-carboline-3-carboxylate* (**8**): The general procedure described in section 3.2.6 was used and the residue was purified by re-crystallization from hot methanol with addition of ethyl acetate (1+5) to give the desired compound in 70% yield, mp 170–172 °C; ^1^H-NMR (DMSO-d_6_): δ 1.96 (1H, bs, N(2)H, c), 2.80 (1H, dd, *J* = 10.2 Hz, C(4)H, c), 3.00 (1H, dd, *J* = 8.4 Hz, C(4)H, c), 3.63 (3H, s, COOCH_3_), 3.71 (3H, s, Ar-OCH_3_), 3.72 (1H, q, *J* = 8.6 Hz, C(3)H, c), 3.84 (1H, s, Ar-OH), 5.10 (1H, q, *J* = 5.4 Hz, C(1)H, c), 6.76 (2H, s, C(5, 6)H, Ph), 6.90 (1H, s, C(2)H, Ph), 6.93~7.41 (4H, m, C(5,6,7,8)H, c), 10.25 (1H, bs, N(9)H, c).

*Methyl 1-(trichloromethyl)-1,2,3,4-tetrahydro-β-carboline-3-carboxylate* (**9**): The procedure described in section 3.2.6 was used and the residue was purified by re-crystallization from ethyl acetate with addition of petroleum ether (1+3) to give **9** in 60% yield, mp > 200 °C. ^1^H-NMR (DMSO-d_6_): δ 2.41 (1H, bs, N(2)H, c), 3.14~3.47 (2H, m, C(4)H, c), 3.62 (3H, s, COOCH_3_), 4.34 (1H, q, *J* = 10.8 Hz, C(3)H, c), 5.16 (1H, q, *J* = 6.8 Hz, C(1)H, c), 7.00~7.45 (4H, m, C(5,6,7,8)H, c), 11.78 (1H, bs, N(9)H, c).

*Methyl 1,2,3,4-tetrahydro-β-carboline-3-carboxylate* (**10**): The method described in section 3.2.6 was used. The residue was purified by column chromatography using methanol+ethyl acetate (1+3) as eluent to produce the desired compound in 50.6% yield, mp > 200 °C; ^1^H-NMR (DMSO-d_6_): δ 3.04 (1H, bs, N(2)H, c), 3.21 (3H, s, COOCH_3_), 3.42 (1H, dd, *J* = 10.5 Hz, C(4)H, c), 3.97 (1H, dd, *J* = 7.8 Hz, C(4)H, c), 4.44 (1H, d, *J =* 7.8 Hz, C(1)H, c), 4.56 (1H, d, *J =* 7.5 Hz, C(1)H, c), 5.47 (1H, q, *J* = 9.4 Hz, C(3)H, c), 7.11~7.53 (4H, m, C(5,6,7,8)H, c), 11.34 (1H, bs, N(9)H, c).

*Methyl 1-methyl-β-carboline-3-carboxylate* (**11**): Methyl 1-methyl-2,3,4,9-tetrahydro-1*H*-pyrido[3,4-b]-indole-3-carboxylate (**6**, 1.3 g) was dissolved in DMF (10 mL) in a round-bottomed, short-necked flask. The solution was cooled to 0 °C and potassium permanganate (0.4 g) was added to the mixture that was then left stirring overnight. The mixture was poured into water to collect the top deposit which was re-crystallized from hot methanol with addition of ethyl acetate(1+1) to give the title compound in 71.3% yield [22], mp > 200 °C; ^1^H-NMR (DMSO-d_6_): δ 2.85 (3H, bs, CH_3_), 4.03 (3H, s, COOCH_3_), 7.37~8.79 (4H, m, C(5,6,7,8)H, c), 9.17 (1H, s, C(4)H, c), 11.62 (1H, bs, N(9)H, c).

*Methyl 1-(4-chlorophenyl)-β-carboline-3-carboxylate* (**12**): The procedure described in section 3.2.6 was used and the reaction product was further purified by re-crystallization from hot methanol to give compound **12** in 60.2% yield, mp > 200 °C; ^1^H-NMR (DMSO-d_6_): δ 3.94 (3H, s, COOCH_3_), 7.34~8.44 (4H, m, C(5,6,7,8)H, c), 7.70~8.05 (4H, m, C(2,3,5,6)H, Ph), 8.96 (1H, s, C(4)H, c), 9.95 (1H, bs, N(9)H, c).

*Methyl 1-phenyl-β-carboline-3-carboxylate* (**13**): The methodology as described in section 3.2.6 was used and the reaction product was further purified by re-crystallization from hot methanol to give the target molecule in 82.7% yield, mp > 200 °C; ^1^H-NMR (DMSO-d_6_): δ 4.02 (3H, s, COOCH_3_), 7.33~7.60 (5H, m, C(2,3,4,5,6)H, Ph), 7.65~8.27 (4H, m, C(5,6,7,8)H, c), 8.90 (1H, s, C(4)H, c), 9.04 (1H, bs, N(9)H, c).

*Methyl 1-(2-hydroxyphenyl)-β-carboline-3-carboxylate* (**14**): The procedure described in section 3.2.6 was used. The reaction product was further purified by re-crystallization from hot methanol to give 14 in 35.5% yield, TLC: R_f_: 0.65 (petroleum ether + ethyl acetate, 5 + 1 by volume), mp > 200 °C; ^1^H-NMR (DMSO-d_6_): δ 3.98 (3H, s, COOCH_3_), 5.71 (1H, m, Ar-OH), 7.08~7.41 (4H, m, C(3,4,5,6)H, Ph), 7.63 (4H, m, C(5,6,7,8)H, c), 8.98 (1H, s, C(4)H, c), 10.27 (1H, bs, N(9)H, c).

*Methyl 1-(5-chloro-3-methyl-1-phenyl-1H-pyrazol-4-yl)-β-carboline-3-carboxylate* (**15**): The procedure described in section 3.2.6 was followed and the reaction product was further purified by re-crystallization from hot methanol to give the title compound in 76.9% yield, mp > 200 °C; ^1^H-NMR (DMSO-d_6_): δ 2.34 (3H, s, CH_3_), 7.32~8.44 (4H, m, C(5,6,7,8)H, c), 7.53~7.60 (5H, m, C(2,3,4,5,6)H, Ph), 78.86 (1H, s, C(4)H, c), 11.84 (1H, bs, N(9)H, c).

*1-Methyl-β-carboline-3-carboxylic acid* (**16**): Methyl 1-methyl-β-carboline-3-carboxylate (**11**, 2.3 g, 10 mmol) was dissolved in methanol (3 mL) and heated to reflux in 2 M aqueous sodium hydroxide solution (20 mL) for 2 h. After completion of the reaction the pH of the mixture was adjusted to 8. The precipitate was filtered off and the residue was re-crystallized from hot methanol as a yellow floc, obtained in 97% yield [29], mp > 200 °C; ^1^H- NMR (DMSO-d_6_): δ 2.83 (3H, s, -CH_3_), 7.29~8.35 (4H, m, C(5,6,7,8)H, c), 8.76 (1H, s, C(4)H, c), 10.81 (1H, bs, N(9)H, c), 12.02 (1H, s, COOH).

### 3.3. Bioassays

To evaluate the biological activities of the synthesized compounds, cytotoxic assays were carried out with insect cultured cell line Sf9 from *Spodoptera frugiperda*, insecticidal assays were conducted with the reared colonies of 4 th instar larvae of *Culex pipiens quinquefasciatus* and apterous adults of mustard aphids [*Lipaphis erysimi* (Kaltenbach)]. The cytotoxic and insecticidal activity of harmine (95%, purity, Sigma) was also evaluated against the aforementioned species as positive control. 

#### 3.3.1. Cytotoxicity

Sf9 cells were maintained at 27 °C in TC-199-MK medium supplemented with 10% FCS (vol/vol), 1% L-glutamine 200 mmol/L and penicillin–streptomycin–neomycin solutions (vol/vol) described in Zhong *et al.* [31]. The cell growth inhibition was measured by the MTT method described by Mosman [32]. Cells were seeded in 96-well microtitration plates at the exponential growth phase. Different concentrations of compounds diluted with medium were added at their log-phase growth stage. And 0.2 μL solvent (DMSO) only was added as control (CK). The final concentration of solvent in the cultures assayed was 1%. Compound was solubilized with dimethyl sulfoxide and the first concentration was 1,000 mg/L. The harmine was used as positive control. After 6 h, 12 h and 24 h of treatment, 5 mg/mL MTT was dissolved in PBS and 20 μL of this stock solution was added to the culture cells. After additional 3 h of incubation, the medium was discarded and the 96-well plates were dried in the air. Then 100 μL DMSO were added to dissolve the formazan crystals, and the absorbance was measured at 570 nm by a microplate reader (Spectramex, 190 Molecular Devices Inc., USA) [31]. The cytotoxic effect was expressed as a relative percentage of inhibition calculated as follows:

Cell growth inhibition rate (%) = [(A _control_ –A _treatment_) / A _control_] ×100

#### 3.3.2. Insecticidal activity

##### 3.3.2.1. Mosquito

Mosquito (4th instar larvae of *Culex pipiens quinquefasciatus*) used in the experiments were obtained from cultures at the Guangdong Center for Disease Control and Prevention. Bioactivities of compounds were tested by the mixed pesticide method by the procedure as recommended in the literature [33,34]. The compounds were first dissolved in a mixture of DMSO and acetone at a concentration of 10 g/L and suspended in distilled water with the addition of 0.5% of the commercial surfactant Tween-80 to obtain final concentrations of 1, 10, 50 and 100 mg/L. Twenty early 4th instar *Cx. pipiens quinquefasciatus* were placed into a 20 mL bioassay bowl containing 10 mL of the test solution and incubated at 27 °C. The number of dead larvae was determined at the start of the experiment (0 h), and after 2 h, 12 h and 24 h, respectively. Larvae were considered dead when they were unable to reach the surface of the solution on shaking the cups. Mortality was scored and compared with controls that had been exposed to a diet treated with solvent only. The cumulative mortality data were corrected by Abbott’s formula.

##### 3.3.2.2. Aphis

Mustard aphids (*Lipaphis erysimi* (Kaltenbach)) were reared on mustard leaves. Apterous adult aphids fed on equally vigorous leaves were selected for the experiments. The compounds were first dissolved in DMSO and acetone mixture solvent at a concentration of 10 g/L and suspended in distilled water with 0.5% of emulsifier to obtain final concentrations of 50, 100 and 200 mg/L. The leaf discs with about 30–50 apterous adult aphids were dipped in compound solutions, shaken gently for 3 s, and then taken out. The excess solution was immediately absorbed with a filter paper. Then treated leaf discs were held at 25 ± 1 °C, 50–60% relative humidity. Solvent only was used as the control. The mortality was recorded at 24 and 48 h after the treatment. The mortality was corrected with reference to the control and assessed statistically [33]. 

### 3.4. Data analyses

Experiments were carried out in triplicate and data analyses were run on the SAS 8.01 (2000) software and P ≤ 0.05 was considered to be significant. Data were subjected to one-way ANOVA and mean values were compared using Duncan’s multiple-range test [35]. Mean values followed by the same letter have no significant difference (P ≤ 0.05) according to Duncan’s multiple-range test.

## 4. Conclusions

Recently, a number of investigations have demonstrated that harmine derivatives not only possessed antifungal activity, but that they were also highly cytotoxic against several kinds of cancer cell lines. However, the insecticidal activity of harmine derivatives has not been previously reported. Due to their diverse biological activities and the feasibility of their simple structure modification, researchers have been strongly interested in the synthesis and the structure-activity relationships of a series of harmine derivatives, especially for the purpose of developing anticancer drugs. In this paper, the preparation of 16 harmine derivatives was reported and their cytotoxicities and insecticidal activities were tested. The compounds **2** and **13** showed the strongest activities both *in vitro* and *in vivo*. They both caused 71% mortality to Sf9 cells after 24 h treatment at a concentration of 200 mg/L. Furthermore, the LC_50_ values for compounds **2** and **13** were 20.82 mg/L and 23.98 mg/L against 4th instar larvae mosquito (*Cx. pipiens quinquefasciatus*) after 24 h treatment, and 53.16 mg/L and 68.05 mg/L against mustard aphids (*L. erysimi*) after 48 h treatment, respectively. The insecticidal activities *in vivo* were in line with the *in vitro* cytotoxicity. These results may provide a basis for the production of novel potential pesticides based on harmine and norharman natural products. Therefore, further in-depth studies to identify the insecticidal mechanism of compounds **2** and **13** are warranted for their development as biorational pesticides.

## Figures and Tables

**Table 1:**
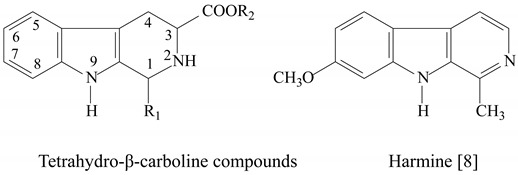
Structure of tetrahydro-β-carboline compounds.

Compound	R_1_	R_2_
1	methyl	-H
2	phenyl	-H
3	-H	-H
4	trichloromethyl	-H
5	3-methoxy-4-hydroxyphenyl	-H
6	methyl	methyl
7	phenyl	methyl
8	3-methoxy-4-hydroxyphenyl	methyl
9	trichloromethyl	methyl
10	-H	methyl

**Table 2:**
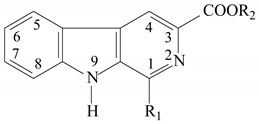
Structure of β-carboline compounds.

Compound	R_1_	R_2_
11	methyl	methyl
12	4-chlorophenyl	methyl
13	phenyl	methyl
14	2-hydroxyphenyl	methyl
15	1-phenyl-3-methyl-5-chloropyrazol-4-yl	methyl
16	methyl	-H
